# Preservice teachers’ teaching internship affects professional identity: Self-efficacy and learning engagement as mediators

**DOI:** 10.3389/fpsyg.2022.1070763

**Published:** 2022-11-30

**Authors:** Zhiling Cai, Jinxing Zhu, Saiqi Tian

**Affiliations:** College of Education, Wenzhou University, Wenzhou, China

**Keywords:** preservice teachers, teaching internship, professional identity, self-efficacy, learning engagement

## Abstract

Preservice teachers’ professional identity is a critical factor in their motivation, effectiveness, and retention. Teaching internship can promote the transformation of professional identity and self-efficacy of preservice teachers. The internship environment also enhances students’ increasing level of learning engagement. Although much research has shown strong relationship between preservice teachers’ teaching internship and professional identity, less is known about how self-efficacy and learning engagement mediated those variables. The purpose of this study is to investigate the roles that self-efficacy and learning engagement play in the relationship between preservice teachers’ professional identities and their teaching internship experiences. The study involved 309 preservice teachers in total, and the variables were measured using questionnaires. For the data analysis, we used structural equation modeling. The study’s findings are as follows. (a) Teaching internship, professional identity, self-efficacy, and learning engagement were all significantly correlated with one another. (b) Both self-efficacy and learning engagement partially mediated the relationship between teaching internship and professional identity in a parallel manner. (c) Self-efficacy and learning engagement also sequentially mediated the relationship between teaching internship and professional identity.

## Introduction

The professional identity of a teacher is individual self-knowledge in teaching-related situations and relationships that reflects in its teaching activities, feelings of belonging, and learning experiences (Ibarra, 1999; Graven and Lerman, 2003). It predicts teachers’ dedication to professional preparation and is a critical factor in their motivation, effectiveness, and retention (Hong, 2010; Timoštšuk and Ugaste, 2010; Huang and Wang, 2021). Preservice teachers’ cognition of the teaching profession is more based on teachers as students (Flores and Day, 2006; Levin and He, 2008; Hong, 2010). It is a long-term process to develop preservice teachers’ professional identity, and in most cases, the transition of identity is difficult (Zhao and Zhang, 2017). However, it has been demonstrated that teaching internship can promote the transformation of professional identity (Izadinia, 2016). For example, Izadinia (2016) verified the effective influence of mentoring relationships on professional identity. Mentor teachers’ support in the internship has a positive effect on student teachers’ decision whether to stay in the teaching profession or leave it. Their construction of self-identification as teachers can also occur *via* the daily classroom and teaching interaction with students (Yuan and Lee, 2015).

Moreover, there were numerous studies investigating changes in professional identity after teaching internship (Leavy et al., 2007; Katz et al., 2011; Zhao and Zhang, 2017; Guimaraes and Costa, 2022). For instance, Zhao and Zhang (2017) investigated 98 preservice teachers by questionnaires and interviewed 12 teachers, and found that after the internship, preservice teachers’ professional identity was enhanced, specifically intrinsic value identity. Another study determined the positive influence of the mentor’s support in the internship on the development of student teachers’ identity (Michos et al., 2022). Many studies have focused on the direct impact of teaching internship on preservice teachers’ professional identity. Few studies have addressed the indirect effect of teaching internship on preservice teachers’ professional identity. In addition, little work has examined the effects of teaching internship on preservice teachers’ professional identity relative to another factor such as learning engagement and self-efficacy. This study, therefore, focuses on the direct and indirect effect of teaching internship on preservice teachers’ professional identity. The present study proposed a theoretical model in which the relationship between teaching internship and professional identity is mediated by self-efficacy and learning engagement. We tested the mediating role of self-efficacy and learning engagement in the relationship between teaching internship and professional identity, and determined how self-efficacy and learning engagement affiliation work together (i.e., in parallel, sequentially, or both) in the above pathway.

## Literature review and hypotheses

Internships are “an ongoing placement of an enrolled student in an organization for a period of time — sometimes paid, sometimes without pay — with a faculty supervisor, a company supervisor, and some academic credit obtained associated with a degree” (Narayanan et al., 2010). In teacher education, teaching internship work-based learning experience is broadly referred to as “teaching practicum” in some countries, sometimes “student teaching” or “teaching practice” (Huu Nghia and Tai, 2019). During the process of teaching internship, preservice teachers have a chance to apply theoretical knowledge and develop practical teaching skills in a real classroom setting (Westerman, 1991). After being trained in a normal college, preservice teachers should be exposed to real teaching situations, apply the obtained pedagogical knowledge and skills, connect with professional teachers, and explore the possibilities of career development (Cohen et al., 2013). In addition, immersion in an experienced teacher practice community provides many opportunities for preservice teachers to develop their teacher identity (Nghia and Tai, 2017) and fill gaps in their professional knowledge as well as skills (Cohen et al., 2013). Many articles have suggested that the teaching internship offers preservice teachers valuable professional development opportunities. For example, although preservice teachers have found their teacher identity before the university, their images or expectations of teachers were nearly modified during the teaching internship (Tran Le Huu and Huynh Ngoc, 2017). Experiences in teaching internship can help them adjust to unrealistic expectations of the profession. A qualitative study found that the internship period was the most valuable and helpful for a preservice teacher in preparation for being a teacher (Salazar Noguera and McCluskey, 2017). Teaching practicum arouses their awareness of vocational identity as teachers, and they begin to realize the transition from being undergraduates to being “teachers.” Accordingly, the first hypothesis of the study is:

Hypothesis (H1): Teaching internship positively influences professional identity.

Some research demonstrated that teaching internship affected preservice teachers’ beliefs (Lacorte, 2009; Li, 2011). During the practicum, their beliefs experience processes of change, including confirmation, realization, disagreement, elaboration, integration, and modification (Yuan and Lee, 2014). After analyzing video recordings of 48 preservice elementary teachers, Kruse et al. (2021) found that teaching practice indirectly impacted self-efficacy. Compared to coursework in teaching and supervised teaching experiences, a teaching internship is the most significant for supporting doctoral students’ development as teachers and is closely related to teaching self-efficacy (Suddeath et al., 2020). During the teaching internship, student teachers are exposed to different ideas and understandings of teaching from dialogic reflection with their mentor, which can also promote their self-efficacy (Stuart and Thurlow, 2000). Studies have shown that self-efficacy is related to and might promote individuals’ professional identity (Moslemi and Habibi, 2019; Qiu et al., 2019; Chen et al., 2020; Eren and Sonay Turkmen, 2020). For example, Canrinus et al. (2012) collected data from 1,214 Dutch teachers and proposed a structural equation model, to investigate the relevant indicators of teachers’ sense of their professional identity. They found that teachers’ classroom self-efficacy and relationship satisfaction affected their sense of professional identity. They highlighted that this presented model could be well applied to other professionals’ sense of their professional identities, such as novice teachers and preservice teachers. Therefore, the hypotheses are formulated as follows:

Hypothesis (H2): Teaching internship positively influences self-efficacy.

Hypothesis (H3): Self-efficacy positively influences professional identity.

Literature suggests that internship has an impact on undergraduates’ learning engagement (Miller et al., 2011). Okolie et al. (2021) emphasized that the association between internship and learning engagement was positive and statistically significant. The internship environment may enhance students’ increasing level of engagement. This would lead to the achievement of career-related goals (Lent et al., 1994), further influencing professional identity. Yu et al. (2021) validated the professional identity model and confirmed the significant correlation between undergraduates’ professional identity and learning engagement. Self-efficacy is also an essential predictor of learning engagement. For instance, a study collected data from 1,930 medical students and found a positive relationship between the participating students’ levels of learning engagement and self-efficacy (Wu et al., 2020). Another research analyzed daily diary questionnaires, seven daily end-of-class computer skills examinations and questionnaires of 121 participants, and found that computer self-efficacy was positively associated with learning engagement (Chen, 2017). Self-efficacy also showed significant positive relationships with learning engagement in online courses (Heo et al., 2021; Kuo et al., 2021; Wang et al., 2022). Consequently, the following hypotheses are proposed:

Hypothesis (H4): Teaching internship positively influences learning engagement.

Hypothesis (H5): Self-efficacy positively influences learning engagement.

Hypothesis (H6): Learning engagement positively influences professional identity.

Considering the role of self-efficacy and learning engagement in preservice teachers’ professional identity, we connected preservice teachers’ teaching internship to their professional identity *via* self-efficacy and learning engagement:

Hypothesis (H7): The relationship between teaching internship and professional identity is mediated by self-efficacy.

Hypothesis (H8): The relationship between teaching internship and professional identity is mediated by learning engagement.

Hypothesis (H9): Self-efficacy and learning engagement play a chain mediating effect between teaching internship and professional identity.

**FIGURE 1:**
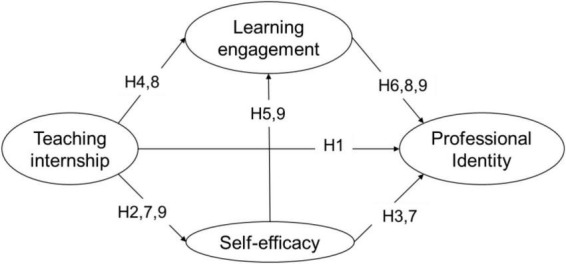
A theoretical model of impacts of preservice teachers’ teaching internship on professional identity.

Figure 1 presents the theoretical model of the hypothesized relationships among preservice teachers’ teaching internship, self-efficacy, learning engagement, and professional identity.

## Methodology

### Research design

In this study, we utilized a correlational research design to describe relationships among quantitative variables (Fraenkel et al., 2011). The dependent variable was professional identity. Independent variables were teaching internship, learning engagement, and self-efficacy.

### Sample of the study

The present study was carried out with 309 preservice teachers. Of the participants, 91.9% was females (*N* = 284) and 8.1% was males (*N* = 25). The gender imbalance among primary and secondary school teachers has become a global trend (Organisation for Economic Co-operation and Development [OECD], 2020). The proportion of female teachers is significantly higher than that of male teachers in China, and the educational profession has become a feminized sector (Zhang, 2018). As a result, only a limited number of male preservice teachers participated in this study. Among them, 31.7% (*N* = 98) had completed the first internship, 35.3% (*N* = 109) had completed the second internship, and 33.0% (*N* = 102) had completed the third internship. During the teaching internship, all the participants were trained in teaching knowledge and skills. The study was approved by the Ethics Committee of College of Education, Wenzhou University. Before the survey, all the participants were informed that their personal information would be anonymous, and they had a right to refuse this participation. It took participants approximately 10 min to complete the scales in a quiet classroom under the supervision of a trained teacher. All questionnaires were written in Chinese. The data were collected from May 2022 to June 2022.

### Data collection instrument

#### Teaching internship

A questionnaire (Supplementary Table 1) which acquired the environment, guidance, assessment, and feeling in teaching internship, was modified from previous reports (Sun and van Es, 2015; Kokkinos et al., 2016; Kartal and Başarmak, 2022). This questionnaire had 15 items under four constructs: environment (three items, e.g., My school has good hardware facilities), guidance (two items, e.g., During the teaching internship, my mentors often instructed me to prepare my class), assessment (four items, e.g., During the teaching internship, my mentors evaluated and examined my performance), and feeling (six items, e.g., I felt happy during the teaching internship). All items were responded on a 5-point Likert-type scale, where 1 = strongly disagree, 2 = disagree, 3 = neutral, 4 = agree, and 5 = strongly agree. Cronbach’s alpha coefficient for the total scale was 0.901. For the present study, internal reliabilities were satisfactory: (a) Environment, α = 0.832, (b) Guidance, α = 0.891, (c) Assessment, α = 0.784, (d) Feeling, α = 0.802. Reliabilities of these subscales were sufficient.

#### Self-efficacy

The reservice teachers’ self-efficacy scale was modified (Arsal, 2014; Choi and Lee, 2018) and administered. This scale includes 15 items rated on a Likert-type scale ranging from 1 (strongly disagree) to 5 (strongly agree). The sub-dimensions are as follows: self-efficacy for instructional strategies, self-efficacy for classroom management, and self-efficacy for student engagement. The whole scale showed acceptable reliability, Cronbach α = 0.961, as did each dimension (Cronbach α = 0.929, 0.876, and 0.891, respectively). For more details, see Supplementary material (Supplementary Table 2).

#### Learning engagement

Utrecht work engagement scale for students (UWES-S) was used to assess student teachers’ learning engagement (Schaufeli et al., 2002). The scale consists of 14 items distributed across the three dimensions: vigor, dedication, and absorption. Learning engagement was rated on a 7-point Likert scale ranging from 0 = never to 7 = always. Reliability coefficients (Cronbach’s α) were 0.962 for the total scale. Internal reliabilities for the three dimensions of burnout were 0.949 for vigor, and 0.944 for dedication. For more details, see Supplementary material (Supplementary Table 3).

#### Professional identity

We measured preservice teachers’ professional identity using a previously validated scale (Zhang et al., 2016) with a Likert self-report scale ranging from 1 (strongly disagree) to 5 (strongly agree). It has three dimensions: intrinsic value identity (Sample item is: “I think it’s happy for a teacher to communicate with students.”), extrinsic value identity (Sample item is: “Teacher is a highly respected occupation.”), and volitional behavior identity (Sample item is: “I often actively participate in trainings and lectures for teacher and teaching for promotion.”). The higher the score, the more professional identity the preservice teachers had. Cronbach alpha’s value for professional identity was found to be 0.943 in the present study, and Cronbach alpha’s values for subscale were 0.923, 0.853, and 0.816. For more details, see Supplementary material (Supplementary Table 4).

### Data analysis

Means (M) and standard deviations (SD) were generated for each subscale. To explore the associations among the variables, correlational analyses were conducted using Pearson’s *r* (Arndt et al., 1999; Hauke and Kossowski, 2011). Structural equation modeling (SEM) was utilized to test the hypothesized models. Model fit was evaluated using a combination of criteria: the normed Chi-square statistic (χ^2^/*df*), comparative fit index (CFI), root mean square error of approximation (RMSEA), and the standardized root mean square residual (SRMR) (Hu and Bentler, 1999). The mediation effects were tested by percentile bootstrap (Preacher and Hayes, 2008). All statistical analyses were run by an online data analysis platform SPSSAU.^1^

## Findings

### Descriptive statistics and correlations

Table 1 presents the descriptive statistics and bivariate correlations between variables under study. More descriptive statistics and bivariate correlations between sub-dimensions of variables are summarized in Supplementary Table 5. The mean scores are higher than the medians in teaching internship and professional identity. But the mean scores of preservice teachers’ self-efficacy and learning engagement are lower than the median. Overall, the results of the bivariate correlations are consistent with our expectations. There are significant relationships between all variables.

**TABLE 1:** Bivariate correlations of study variables and descriptive statistics.

Variables	M	SD	Median	1	2	3
1. Teaching internship	4.098	0.615	4.067			
2. Self-efficacy	3.870	0.658	3.933	0.577**		
3. Learning engagement	5.344	1.104	5.429	0.635**	0.643**	
4. Professional identity	4.003	0.655	4.000	0.647**	0.659**	0.765**

**p < 0.01.

### Measurement model

**FIGURE 2:**
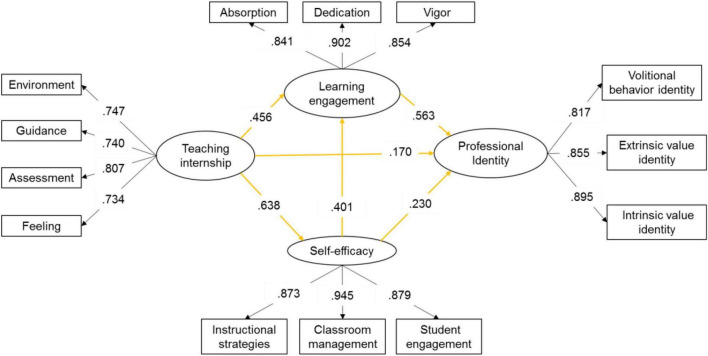
Standardized results for the measurement model. All the item parameter estimates and the latent variable correlations were significant at *p* < 0.01.

The structural equation model analyses are depicted in Figure 2. The CFA results of the measurement model reveal an acceptable model fit [(χ^2^) = 295.577; χ^2^/*df* = 5.01; *p* < 0.001; CFI = 0.912; RMSEA = 0.114; SRMR = 0.182]. All the item parameter estimates and the latent variable correlations are significant at *p* < 0.001.

The direct effects among variables are summarized in Table 2. The paths from teaching internship to professional identity (β = 0.170, *p* < 0.01), self-efficacy (β = 0.638, *p* < 0.01), and learning engagement (β = 0.456, *p* < 0.01), are statistically significant. Results reveal that teaching internship positively influences learning engagement, self-efficacy, and professional identity (H1, H2, and H4). The path from self-efficacy to learning engagement is statistically significant (β = 0.401, *p* < 0.01). Also, teaching internship positively influences learning engagement (H5). The paths from learning engagement (β = 0.563, *p* < 0.01) and self-efficacy (β = 0.230, *p* < 0.01) to professional Identity are both statistically significant. Accordingly, learning engagement and self-efficacy positively influence professional identity (H3 and H6).

**TABLE 2:** Direct effects among variables.

Factor	→	Variables	*B*	β	SD	Z	p
Teaching internship	→	Professional identity	0.181	0.170	0.068	2.657	0.008**
Teaching internship	→	Self-efficacy	0.660	0.638	0.066	9.962	0.000**
Self-efficacy	→	Professional identity	0.237	0.230	0.060	3.959	0.000**
Teaching internship	→	Learning engagement	0.726	0.456	0.109	6.676	0.000**
Self-efficacy	→	Learning engagement	0.616	0.401	0.097	6.361	0.000**
Learning engagement	→	Professional identity	0.377	0.563	0.049	7.740	0.000**

**p < 0.01.

### Mediation model

In order to test mediation effects (H7, H8, and H9), the indirect effect is computed in each of these bootstrap samples (Chen et al., 2014). Table 3 displays the indirect effect process and indirect effects as well as their associated 95% confidence intervals (CIs). All indirect effects differed significantly from zero (Preacher and Hayes, 2008). As a result, teaching internship exerts an indirect effect on professional identity *via* self-efficacy (H7). Teaching internship exerts an indirect effect on professional identity *via* learning engagement (H8). Thus, self-efficacy and learning engagement parallelly mediate the link between teaching internship and professional identity. Furthermore, self-efficacy and learning engagement play a chain mediating effect between teaching internship and professional identity (H9). In summary, self-efficacy and learning engagement mediate the association between teaching internship and professional identity not only parallelly but also sequentially. There is partial mediation for the path from teaching internship to professional identity.

**TABLE 3:** Standardized indirect effects and 95% confidence intervals.

	Model pathways	Effect	95% CI	
			Lower	Upper
Indirect	TI→SE	0.302	0.188	0.416
effect process	TI→LE	1.141	0.986	1.296
	SE→LE	0.276	0.213	0.340
	SE→PI	0.225	0.136	0.314
	LE→PI	0.290	0.234	0.347
Indirect	TI→SE→PI	0.068	0.020	0.127
effects	TI→LE→PI	0.331	0.216	0.405
	TI→LE→SE→PI	0.071	0.025	0.105

TI, Teaching internship; LE, Learning engagement; SE, Self-efficacy; PI, Professional identity. All the path coefficients are significant at the 0.01 level.

## Discussion and conclusion

This study provided evidence for the relationship between preservice teachers’ teaching internship and professional identity through the mediating role of self-efficacy and learning engagement. Based on the literature, a model was proposed and tested empirically. Using the data of 309 preservice teachers, we constructed a final model with satisfactory fit indices incorporating all indicators. In line with our expectations, the teaching internship is, directly and indirectly, related to preservice teachers’ professional identity. Preservice teachers’ self-efficacy and learning engagement not only parallelly but also sequentially mediate the relationship between teaching internship and professional identity.

### Teaching internship and professional identity

As for Hypothesis 1, results indicate that teaching internship is positively associated with professional identity. This means that better teaching internship can increase preservice teachers’ professional identity. As a dynamic process, professional identity can be changed along with teachers’ growth (Geijsel and Meijers, 2005; Korthagen and Vasalos, 2005). The environment of the internship, guidance by mentors, assessment, and feeling in teaching internship can influence a preservice teacher’s professional identity. We also found that preservice teachers’ intrinsic value identity is relatively higher than extrinsic value identity and volitional behavior identity after teaching internship. Intrinsic value identity, mainly related to one’s subjective feelings toward the teaching profession, is closely affected by feelings in the teaching internship. These findings are congruent with the results of previous studies (Katz et al., 2011; Zhao and Zhang, 2017; Wei et al., 2021; Guimaraes and Costa, 2022).

In light of the direct effect of teaching internship on professional identity, teacher educators should provide more opportunities for preservice teachers to participate in multiform practical work to actively and reflectively build a professional identity (Richter et al., 2021). Mentor plays an important role, not only directly influencing preservice teachers’ professional identity but also identity affecting their future professional commitment (Schepens et al., 2009; Christophersen et al., 2016; Izadinia, 2016). Accordingly, Zhao and Zhang (2017) proposed that proficient mentors should be arranged for preservice teachers. It is also of great necessity to make assessments for preservice teachers from different angles (McKellar et al., 2020).

### Self-efficacy and learning engagement as mediators

Self-efficacy partially mediates the association between teaching internship and professional identity (H7). This result echoes previous empirical research by Canrinus et al. (2012), Dalioglu and Adiguzel (2016), Topkaya (2016), and Michos et al. (2022). During the teaching internship, preservice teachers are involved in a real teaching situation in the classroom, able to bridge the teacher education curriculum with the school context, and organized to reflect on their teaching experiences (Michos et al., 2022). Their self-efficacy has been identified to be strengthened after teaching practice (Dalioglu and Adiguzel, 2016; Topkaya, 2016). Preservice teachers with higher self-efficacy usually have higher relationship satisfaction, and this would improve their sense of professional identity (Canrinus et al., 2012). Other studies reported no significant correlation between self-efficacy and science teaching practices (Kruse et al., 2021). The authors declared that self-efficacy might not be directly related to teaching practices in the population of preservice elementary teachers. This is probably a good explanation of why self-efficacy partially mediates the association between teaching internship and professional identity, rather than fully mediates the association.

Learning engagement partially mediates the association between teaching internship and professional identity (H8). Teaching internship could promote preservice teachers’ professional identity directly and indirectly through their learning engagement. When preservice teachers have more opportunities to take internships, they are more likely to be engaged in learning tasks. More learning engagement would lead to a more robust professional identity. Research studies (Miller et al., 2011; Mancini et al., 2015; Tang, 2020; Yu et al., 2021) indicate similar findings.

The findings of this study also indicate both parallel and sequential mediation *via* self-efficacy and learning engagement on the relationship between teaching internship and professional identity (H9). Although related literature includes some studies indicating the relationship between teaching internship and professional identity (Katz et al., 2011; Zhao and Zhang, 2017; Chua et al., 2018; Guimaraes and Costa, 2022), the studies examining the mediation effect on this relationship are very rare. On the one hand, teaching internship is significantly associated with self-efficacy and learning engagement, both of which impact professional identity. On the other hand, the impact of teaching internship on professional identity is sequentially mediated through self-efficacy and learning engagement.

On the basis of these mediating effects, we suggest that it is necessary to strengthen preservice teachers’ professional identity by promoting self-efficacy and learning engagement in teaching internship. According to Bandura (1977, 1995), an individual’s self-efficacy can be aroused by master experiences, modeling by others, and verbal persuasion. Besides, Skinner’s self-system model of motivational development (SSMMD) highlighted the influence of environmental perception and emotion on learning engagement (Skinner et al., 2008). Consequently, teacher candidates are encouraged to apply professional knowledge to solve the difficulties in real schools. They should be provided more chances to communicate with each other and exchange feelings as well as experiences among both themselves and between students and in-service teachers (Yuan and Lee, 2015). Setting good examples is also essential to help them refine their modeling of outstanding teachers. Prior to, during, and after the teaching internship, self-reflection and journaling, and constructive feedback should also be requested (Cohen, 2010).

### Limitations and future research

Naturally, there are still limitations in this study that can be improved in future research. First, the participants were only recruited from one university in China, and therefore, the sample cannot represent all preservice teachers. Caution should be made when generalizing the results among all preservice teachers. A stratified random sampling method can be used to elicit data from diverse universities to obtain a more representative sample. Secondly, due to the limited sample, this study did not distinguish demographic information (such as gender and age) and educational background (such as major and grade) in analyzing the relationship between teaching internship and professional identity. Whether demographic information and educational background influence professional identity need further investigation. Finally, we only used questionnaires or quantitative data to construct the model in this paper. Future studies will attempt to use interview methods, observation methods, or add other qualitative data. In these ways, in-depth information about preservice teachers’ professional identity, self-efficacy, learning engagement, and other aspects in teaching internship could be gathered.

## Data availability statement

This original contributions presented in this study are included in the article/Supplementary material, further inquiries can be directed to the corresponding author.

## Author contributions

ZC and ST: conceptualization, methodology, and writing—review and editing. ZC: validation and writing—original draft preparation. JZ: formal analysis and investigation. ST: supervision. All authors contributed to the article and approved the submitted version.

## Footnotes

1
https://spssau.com/
