# Supplementary material for: Preservice teachers’ teaching internship affects professional identity: Self-efficacy and learning engagement as mediators
Source: Front Psychol. 2022 Nov 30;13:1070763. doi: 10.3389/fpsyg.2022.1070763 (PMC9748549; doi:10.3389/fpsyg.2022.1070763)
Supplement: Supplementary file 1 [file Data_Sheet_1.docx]

Supplementary Material

**Preservice teachers’ teaching internship affects professional identity: self-efficacy and learning engagement as mediators**

Zhiling Cai, Jinxing Zhu, Saiqi Tian^*^

College of education, Wenzhou University, Wenzhou, China

^*^tiansaiqi@wzu.edu.cn

Table S1. Descriptive statistics of teaching internship

| No. | Items | SA (%) | A (%) | U (%) | D (%) | SD (%) | Dimensions |
| --- | --- | --- | --- | --- | --- | --- | --- |
| 1 | My school has good hardware facilities | 36.9 | 43.0 | 11.7 | 6.8 | 1.6 | a |
| 2 | I can take part in all kinds of teaching activities | 38.5 | 40.8 | 14.2 | 5.2 | 1.3 | a |
| 3 | I can take part in teaching activities on my own terms. | 38.8 | 43.7 | 11.3 | 4.5 | 1.6 | a |
| 4 | During the teaching internship, my mentors often instructed me to prepare my class. | 47.6 | 35.0 | 10.4 | 5.8 | 1.3 | b |
| 5 | During the teaching internship, my mentors often instructed me for class management. | 47.6 | 35.0 | 11.0 | 5.2 | 1.3 | b |
| 6 | During the teaching internship, my mentors evaluated my performance. | 48.2 | 41.7 | 6.8 | 2.3 | 1.0 | c |
| 7 | During the teaching internship, other preservice teachers evaluated my performance. | 36.9 | 45.6 | 10.4 | 5.5 | 1.6 | c |
| 8 | During the teaching internship, my students evaluated my performance. | 27.2 | 39.2 | 23.0 | 8.1 | 2.6 | c |
| 9 | During the teaching internship, I evaluated my performance. | 44.0 | 51.5 | 3.9 | 0.0 | 0.6 | c |
| 10 | I felt happy during the teaching internship. | 33.7 | 42.1 | 17.5 | 4.2 | 2.6 | d |
| 11 | I can feel a sense of belonging in the class, which makes me feel warm. | 39.5 | 39.2 | 12.9 | 7.1 | 1.3 | d |
| 12 | My behavior had a positive effect on my students. | 41.4 | 39.2 | 12.6 | 5.8 | 1.0 | d |
| 13 | Teaching internships can help me achieve more in the future. | 34.3 | 35.9 | 20.4 | 6.1 | 3.2 | d |
| 14 | Teaching Internship is a waste of time. | 4.2 | 7.1 | 7.1 | 32.7 | 48.9 | d |
| 15 | I prefer to study in university rather than take part in a teaching internship. | 5.5 | 10.0 | 17.5 | 33.3 | 33.7 | d |

*Note: SD= SA =Strongly Agree, A= Agree, U= Uncertain, D = Disagree, Strongly Disagree. Dimensions: a, environment; b, guidance; c, assessment; d, Feeling.*

Table S2. Descriptive statistics of self-efficacy.

| No. | Items | SA (%) | A (%) | U (%) | D (%) | SD (%) | Dimensions |
| --- | --- | --- | --- | --- | --- | --- | --- |
| 1 | I can identifying the objectives and content of teaching. | 25.243 | 53.074 | 18.447 | 2.913 | 0.324 | a |
| 2 | I can select the teaching methods according to the objectives | 22.654 | 46.926 | 25.89 | 4.207 | 0.324 | a |
| 3 | I can use multiple methods in teaching | 21.359 | 43.689 | 29.773 | 4.531 | 0.647 | a |
| 4 | I can use instructional materials in teaching effectively | 23.301 | 49.191 | 23.301 | 3.56 | 0.647 | a |
| 5 | I can motivate students to learn subjects. | 19.417 | 41.1 | 35.275 | 3.56 | 0.647 | a |
| 6 | I can connect between new knowledge and previous experiences of students. | 21.036 | 44.984 | 28.155 | 5.502 | 0.324 | a |
| 7 | I can plann teaching activities according to the readiness of students. | 20.065 | 42.395 | 29.126 | 7.767 | 0.647 | a |
| 8 | I can plan student-centered teaching activities. | 21.683 | 49.838 | 23.301 | 4.854 | 0.324 | a |
| 9 | I can reinforce correct behaviours of students. | 24.919 | 49.515 | 22.977 | 1.942 | 0.647 | c |
| 10 | I can provide feedback to behaviours of students. | 26.537 | 49.191 | 21.036 | 3.236 | 0 | c |
| 11 | I can correct the negative behaviours of students. | 22.006 | 45.955 | 25.89 | 5.178 | 0.971 | c |
| 12 | I can summarize the course. | 27.832 | 52.751 | 16.828 | 2.265 | 0.324 | b |
| 13 | I can use course time effectively | 19.094 | 45.307 | 30.421 | 3.883 | 1.294 | b |
| 14 | I can create democratic classroom environment. | 24.272 | 49.838 | 22.977 | 2.913 | 0 | b |
| 15 | I can evaluate learning outcomes of students. | 17.476 | 44.984 | 31.392 | 5.825 | 0.324 | b |

*Note: SD= SA =Strongly Agree, A= Agree, U= Uncertain, D = Disagree, Strongly Disagree. Dimensions: a, instructional strategies; b, student engagement; c, classroom management.*

Table S3. Descriptive statistics of learning engagement.

| No. | Items | Never | → | | | | | Alyways | Dimensions |
| --- | --- | --- | --- | --- | --- | --- | --- | --- | --- |
| 1 | When I’m studying, I feel mentally strong. | 17.8 | 23.3 | 29.1 | 19.1 | 5.8 | 3.9 | 1.0 | a |
| 2 | When I get up in the morning, I feel like going to class. | 16.8 | 22.3 | 20.4 | 22.7 | 9.1 | 5.5 | 3.2 | a |
| 3 | I can continue for a very long time when I am studying. | 15.2 | 22.7 | 20.4 | 24.3 | 9.4 | 5.2 | 2.9 | a |
| 4 | When I study, I feel like I am bursting with energy. | 15.5 | 20.4 | 22.3 | 22.7 | 10.0 | 5.8 | 3.2 | a |
| 5 | When studying I feel strong and vigorous. | 19.4 | 26.5 | 23.3 | 18.1 | 6.8 | 4.5 | 1.3 | a |
| 6 | I find my studies to be full of meaning and purpose. | 29.8 | 35.3 | 21.0 | 8.7 | 3.9 | 0.0 | 1.3 | b |
| 7 | My studies inspire me. | 26.9 | 32.0 | 26.2 | 9.7 | 2.6 | 1.0 | 1.6 | b |
| 8 | I am enthusiastic about my studies. | 24.3 | 32.4 | 24.9 | 11.0 | 4.2 | 1.6 | 1.6 | b |
| 9 | I am proud of my studies. | 29.8 | 28.8 | 27.5 | 8.7 | 2.9 | 0.3 | 1.9 | b |
| 10 | I find my studies challenging. | 38.5 | 32.7 | 19.4 | 7.4 | 1.0 | 0.3 | 0.6 | b |
| 11 | Time flies when I’m studying. | 30.7 | 31.1 | 21.0 | 11.7 | 2.9 | 1.9 | 0.6 | c |
| 12 | When I am studying, I forget everything else around me. | 17.8 | 28.2 | 26.5 | 18.1 | 5.2 | 3.6 | 0.6 | c |
| 13 | I feel happy when I am studying intensively. | 26.5 | 31.1 | 22.0 | 15.2 | 2.9 | 1.3 | 1.0 | c |
| 14 | I can get carried away by my studies. | 16.5 | 23.6 | 32.7 | 18.8 | 5.2 | 2.3 | 1.0 | c |

*Note: SD= SA =Strongly Agree, A= Agree, U= Uncertain, D = Disagree, Strongly Disagree. Dimensions: a, vigor; b, dedication; c, absorption.*

Table S4. Descriptive statistics of professional identity.

| No. | Items | SA (%) | A (%) | U (%) | D (%) | SD (%) | Dimensions |
| --- | --- | --- | --- | --- | --- | --- | --- |
| 1 | I think teacher is a rewarding profession. | 58.9 | 37.9 | 2.9 | 0.3 | 0.0 | a |
| 2 | I think the development space for teacher profession is big. | 44.0 | 39.2 | 12.0 | 4.5 | 0.3 | a |
| 3 | I like teaching. | 36.2 | 44.3 | 15.2 | 3.9 | 0.3 | a |
| 4 | I admire the way the teacher live and work. | 31.1 | 34.3 | 26.9 | 5.8 | 1.9 | a |
| 5 | I think it’s happy for a teacher to communicate with students. | 43.7 | 40.8 | 12.0 | 3.2 | 0.3 | a |
| 6 | I think teacher’s work is very interesting. | 34.6 | 41.1 | 19.7 | 4.2 | 0.3 | a |
| 7 | I think being a teacher is fulfilling/brings sense of achievement. | 49.5 | 39.5 | 8.7 | 2.3 | 0.0 | a |
| 9 | I think the work environment and condition for teacher is great. | 29.1 | 32.7 | 28.5 | 9.4 | 0.3 | b |
| 10 | I think teachers’ social status is high. | 26.2 | 37.9 | 28.2 | 7.1 | 0.6 | b |
| 11 | Teacher is a highly respected occupation | 39.8 | 46.9 | 11.7 | 1.6 | 0.0 | b |
| 12 | I frequently pay attention to the information about teacher profession, including policies on welfare, medical service, and opportunity for advanced courses taking and so on. | 26.5 | 36.6 | 27.8 | 7.8 | 1.3 | c |
| 13 | I often actively participate in trainings and lectures for teacher and teaching for promotion. | 20.7 | 33.7 | 32.4 | 11.7 | 1.6 | c |
| 14 | I often read books that related to teacher and teaching. | 19.1 | 32.4 | 31.1 | 15.9 | 1.6 | c |
| 15 | Even if there is no policy constraint (e.g., future job assigning), I will still choose to be a teacher. | 26.2 | 42.4 | 24.9 | 5.8 | 0.6 | c |
| 16 | I think teacher is a rewarding profession. | 58.9 | 37.9 | 2.9 | 0.3 | 0.0 | a |

*Note: SD= SA =Strongly Agree, A= Agree, U= Uncertain, D = Disagree, Strongly Disagree. Dimensions: a, intrinsic value identity; b, extrinsic value identity; c, volitional behavior identity*

Table S5. Descriptive statistics of teaching internship

| Dimensions | M | SD | 1.1 | 1.2 | 1.3 | 1.4 | 2.1 | 2.2 | 2.3 | 3.1 | 3.2 | 3.3 |
| --- | --- | --- | --- | --- | --- | --- | --- | --- | --- | --- | --- | --- |
| 1.1 Environment | 4.101 | 0.799 |  |  |  |  |  |  |  |  |  |  |
| 1.2 Guidance | 4.220 | 0.887 | 0.687** |  |  |  |  |  |  |  |  |  |
| 1.3 Assessment | 4.062 | 0.719 | 0.620** | 0.666** |  |  |  |  |  |  |  |  |
| 1.4 Feeling | 4.015 | 0.729 | 0.523** | 0.496** | 0.620** |  |  |  |  |  |  |  |
| 2.1 Instructional strategies | 3.859 | 0.707 | 0.423** | 0.442** | 0.533** | 0.416** |  |  |  |  |  |  |
| 2.2 Classroom management | 3.853 | 0.684 | 0.399** | 0.409** | 0.557** | 0.438** | 0.824** |  |  |  |  |  |
| 2.3 Student engagement | 3.927 | 0.718 | 0.375** | 0.346** | 0.501** | 0.431** | 0.765** | 0.833** |  |  |  |  |
| 3.1 Vigor | 4.949 | 1.368 | 0.406** | 0.404** | 0.564** | 0.581** | 0.577** | 0.575** | 0.483** |  |  |  |
| 3.2 Dedication | 5.693 | 1.110 | 0.421** | 0.372** | 0.586** | 0.659** | 0.570** | 0.576** | 0.513** | 0.753** |  |  |
| 3.3 Absorption | 5.344 | 1.104 | 0.383** | 0.255** | 0.509** | 0.492** | 0.529** | 0.548** | 0.500** | 0.727** | 0.772** |  |
| 4.1 Intrinsic value identity | 4.172 | 0.667 | 0.440** | 0.333** | 0.540** | 0.636** | 0.523** | 0.612** | 0.592** | 0.641** | 0.704** | 0.596** |
| 4.2 Extrinsic value identity | 3.959 | 0.773 | 0.443** | 0.330** | 0.560** | 0.565** | 0.481** | 0.557** | 0.564** | 0.589** | 0.625** | 0.542** |
| 4.3 Volitional behavior identity | 3.697 | 0.778 | 0.398** | 0.410** | 0.560** | 0.491** | 0.600** | 0.615** | 0.529** | 0.703** | 0.631** | 0.612** |

Note: **= *p* < .001
